# Defining a Patient-Centred Core Outcome Domain Set for the Assessment of Hearing Rehabilitation With Clients and Professionals

**DOI:** 10.3389/fnins.2022.787607

**Published:** 2022-05-03

**Authors:** David Allen, Louise Hickson, Melanie Ferguson

**Affiliations:** ^1^National Acoustic Laboratories, Sydney, NSW, Australia; ^2^Department of Linguistics, Macquarie University, Sydney, NSW, Australia; ^3^School of Health and Rehabilitation Sciences, The University of Queensland, Brisbane, QLD, Australia; ^4^Ear Science Institute Australia, Perth, WA, Australia; ^5^Curtin enAble Institute, Faculty of Health Sciences, Curtin University, Perth, WA, Australia

**Keywords:** outcome assessment (health care), correction of hearing impairment, hearing loss, audiology, patient reported outcome measures (PROMs)

## Abstract

**Background:**

A variety of outcome domains are currently measured for the assessment of hearing rehabilitation. To date, there is no consensus about which outcome domains should be measured, when they should be measured, and how they should be measured. In addition, most studies seeking to develop core outcome sets and measures for hearing rehabilitation services have primarily focussed on the opinions and expertise of researchers, and, to a lesser extent, clinicians, rather than also involving clients of those services. The principles of experience-based co-design suggest that health services, researchers, and policymakers should come together with clients and their families to design health services and define what metrics should be used for their success.

**Objectives:**

This study aimed to seek views and consensus from a range of key stakeholders to define which client-centred self-report outcome domains should be measured, when they should be measured, and how they should be measured, in a national publicly funded hearing rehabilitation scheme. In addition, the study aimed to identify current and future potential mechanisms and systems to standardise the collection of data and reporting of outcomes, to enable comparison across clients and hearing service providers.

**Methods:**

Two stakeholder groups participated in a three-round online Delphi process: (1) 79 professional stakeholders involved in the delivery of hearing services in Australia, and (2) 64 hearing rehabilitation services’ clients identified by not-for-profit consumer organisations. An initial set of in-person workshops scoped the key issues upon which to develop the initial open-ended questions and subsequent Likert-scale statements addressing these issues. These statements were then distributed to both groups in an online survey. The respondent ratings were summarised, and the summary was returned to respondents along with a second round of the survey. This process was then repeated once more. The five most important outcome domains from both groups were then combined, and a consensus workshop of seven professionals and three client advocates agreed on the top four ranked domains.

**Results:**

A range of potential outcome domains were identified as relevant indicators of successful hearing rehabilitation. Communication ability, personal relationships, wellbeing, and participation restrictions were identified as a core outcome domain set that should be measured as a minimum for patients receiving hearing rehabilitation. There was little agreement on the preferred timepoints for collection of outcome measures, with respondents expressing the view that this should be established by research once a set of outcome measures has been selected. However, there was broad agreement that measurements of these domains should be collected at baseline (before the provision of hearing rehabilitation) and no earlier than 3 months following the completion of rehabilitation. Potential benefits and issues with the development of a national outcomes database/collection system were also identified and prioritised, with participants highlighting the importance of valid, high-quality, trustworthy, and comprehensive data collection.

**Conclusion:**

These results provide a Core Outcome Domain Set for the self-reported evaluation of hearing rehabilitation and provide important background information for the design of methods to implement them across hearing healthcare systems. However, the wide range of outcome domains identified as potentially providing important additional information and the lack of specific measures to address these domains strongly suggest that there is still more research to be done. Ongoing stakeholder engagement will continue to be vital for future implementation. In addition, further research is required to determine the optimal time following hearing rehabilitation to utilise any particular outcome measure.

## Introduction

Hearing loss is a chronic condition that affects around four million adults in Australia, which represents one in six of the population (Access Economics Pty Ltd, 2015). In addition, hearing loss can have substantial negative consequences, including activity limitations, participation restrictions, stigmatisation, reduced quality of life, and third-party disability (Chia et al., 2007; Wallhagen, 2010; Scarinci et al., 2012; Granberg et al., 2014b; Heffernan et al., 2016; Barker et al., 2017). Furthermore, hearing loss has been associated with depression, cognitive decline, and dementia (Lin, 2011; Dawes et al., 2015).

Auditory rehabilitation aims to address the negative impact of hearing loss and includes a range of interventions. The primary intervention is hearing aids, which have been shown to be clinically effective in terms of listening ability, hearing-related quality of life (i.e., participation) and health-related quality of life (Ferguson et al., 2017). There are other auditory rehabilitation interventions for adults with hearing loss, which include alternative listening devices such as hearables, communication and patient education, and auditory training (Wong and Hickson, 2012; Ferguson et al., 2016; Ferguson et al., 2019). However, systematic reviews on these interventions have identified a lack of high-quality evidence (Henshaw and Ferguson, 2013; Barker et al., 2016; Maidment et al., 2016; Ferguson et al., 2017; Lawrence et al., 2018) in part due to a lack of a “gold standard” outcome measure (Granberg et al., 2014a; Hall et al., 2019).

In order to assess the effectiveness of interventions for adults with hearing loss, irrespective of the intervention type, it is essential to have appropriate and sensitive outcome measures that are relevant to the outcome domains targeted for improvement by auditory rehabilitation (Ferguson and Henshaw, 2015; British Society of Audiology, 2016). These are essential to both measuring an individual’s progress toward desired goals, often as a result of an intervention, as well as evaluating the overall effectiveness of audiology services and providers of hearing healthcare. Careful consideration needs to be given to which outcome measures are most fit for purpose. For example, a measure that asks only about specific pre-determined situations may not be relevant to the individual, and may not be compatible with a goal-setting approach to rehabilitation that is person-centred and focussed on the individual (British Society of Audiology, 2016).

One of the major problems with measuring outcomes within auditory rehabilitation is the large number of tools and instruments, including behavioural and self-report measures (Granberg et al., 2014a). In particular, there are a huge number of self-report measures available, with one study identifying 139 hearing-specific questionnaires (Akeroyd et al., 2015). Another major problem is that there is no agreement amongst researchers and clinicians in the field regarding what outcomes should be measured and how they should be measured (PricewaterhouseCoopers Australia, 2017). A systematic review of outcome measures used in research demonstrated the extent of this problem (Granberg et al., 2014a), identifying 51 self-report outcome measurement instruments used across 122 adult hearing loss studies. Of these 51, only 16 instruments had been used in more than one study. It is perhaps not surprising then that a scoping review uncovered considerable heterogeneity in outcome measurement in randomised controlled trials of adult auditory rehabilitation interventions (Barker et al., 2015a).

Many of these measures measure similar underlying constructs, such as hearing device use, benefit, satisfaction, and social participation. In the context of hearing outcomes, these underlying constructs are known as outcome domains. However, even among outcome domains that are in widespread use and seen to be important indicators of successful rehabilitation, such as hearing aid use, there is little consensus around which outcome measures should be used (Perez and Edmonds, 2012). Furthermore, there is an increasing awareness globally that outcome domains that are not solely associated with hearing aid amplification and that address participation restrictions and psychosocial aspects should also be considered, such as wellbeing, identity, and emotion (Bennett et al., 2018; Heffernan et al., 2018a; Bennett et al., 2020; Vercammen et al., 2020). However, many of the most widely used standardised outcome measures, such as the International Outcomes Inventory for Hearing Aids (IOI-HA; Cox et al., 2000), do not address these broader and more recently identified outcome domains.

The evidence is clear that both auditory rehabilitation clinical practice and research lack a single (or even a few) outcome measure that is used widely and consistently and accepted as a “gold standard” instrument. Furthermore, even though there is a large number and variety of measures within the field, clinical trials of adult auditory rehabilitation interventions have overlooked outcomes such as adverse effects and quality of care that may be important to key stakeholders, especially patients, hearing healthcare professionals and commissioners of hearing healthcare (Ferguson et al., 2017). The involvement of these stakeholder groups in the development of such tools is rare, with some exceptions (Heffernan et al., 2018a; Heffernan et al., 2019), as typically it has been researchers alone who have developed outcomes.

A major consequence of an non-standardised approach to outcome measurement is that comparison across different patient cohorts and services is almost impossible. Similarly, within research, it is very difficult to compare and combine the results of different trials that use different measures (for example in systematic reviews with meta-analyses), which results in reduced relevance of the results and increased risk of outcome reporting bias (Ferguson et al., 2017).

Within the Australian hearing healthcare context, hearing services are provided free of charge to over one million people each year through the Hearing Services Program (HSP), primarily through the Voucher Scheme, at a cost of $590 million per annum (Commonwealth of Australia, 2019). The Voucher Scheme provides subsidised hearing services to eligible pensioners, Veterans, service people, and those receiving support for a disability that places their employment at risk (Department of Health, n.d.). Currently, as is seen in many other countries, standardised use of patient-centred outcome measures is not prevalent in Australian hearing healthcare, and typically outputs such as hearing aid uptake are used to measure the success of hearing aids for both clients and service providers (PricewaterhouseCoopers Australia, 2017). Although the importance of measuring client outcomes is highlighted in the regulatory framework of the HSP, typically the Australian-developed Client Orientated Scale of Improvement (COSI; Dillon et al., 1997) or the IOI-HA are used. While the COSI does involve recipients in the development of personalised items, potentially overcoming this limitation of the IOI-HA, its insensitivity makes it unsuitable for the measurement of service outcomes (Dillon et al., 1999).

A Government-commissioned review of the HSP published in 2017 found that the majority of key healthcare stakeholders (i.e., Contracted Service Providers, Device Manufacturers, consumer groups, research organisations) who were consulted agreed that client outcomes were important, however there was no consensus on how they should be measured (PricewaterhouseCoopers Australia, 2017). Four types of measurement methods were identified as in common use—the COSI, the IOI-HA, hearing aid datalogging, and speech testing—but none of these were used consistently. The recommendations from this review were to (i) move quickly toward an outcomes-based model rather than an outputs-based model (i.e., focussing on the number of rehabilitation programmes delivered and devices fitted), (ii) consult with key stakeholders to achieve a consensus on which outcomes should be used and to standardise the approach to measuring these, and (iii) identify how outcomes could be measured across service providers and client groups.

This current study aimed to identify and standardise a Core Outcome Domain Set (CODS): a set of outcome domains that should be used as a minimum standard for the assessment of a health condition (Hall et al., 2018), as well as when and how these domains should be assessed. A CODS can then form the basis for development of a core outcome set (COS): “an agreed standardised set of outcomes that should be measured and reported, as a minimum, in all clinical trials” (OMET Initiative, n.d.). The development of COSs has grown in stature in over the years and COSs are now a recommended component of clinical trial protocols, Cochrane reviews, and government funding applications (Williamson and Clarke, 2012; Kirkham et al., 2017). Within hearing rehabilitation, a roadmap to develop a COS for tinnitus treatment has been proposed, which stresses that a consensus is needed on *what* outcome domains should be measured, and then *how* this should be measured using an outcomes tool (Hall et al., 2015; Fackrell et al., 2017). The first of these steps is the development of the CODS; it is then the addition of standardised measurement tools that results in an implementable COS. The overall aim of the present study was to achieve the first of these steps to identify a Core Outcome *Domain* Set for self-report within hearing rehabilitation, in the Australian context.

The specific aims of this study were to:

1. Seek views and consensus from a range of key stakeholders to define which client-centred outcome domains should be used, when they should be measured, and how they should be measured, for the assessment of hearing rehabilitation delivered within a national publicly funded hearing rehabilitation scheme.
2. Identify current and future potential mechanisms and systems to standardise the collection of data and reporting of outcomes, to enable comparison across clients and hearing service providers.

## Materials and Methods

This study was approved by the Hearing Australia Human Research Ethics Committee. Informed consent was obtained from all participants.

**FIGURE 1:**
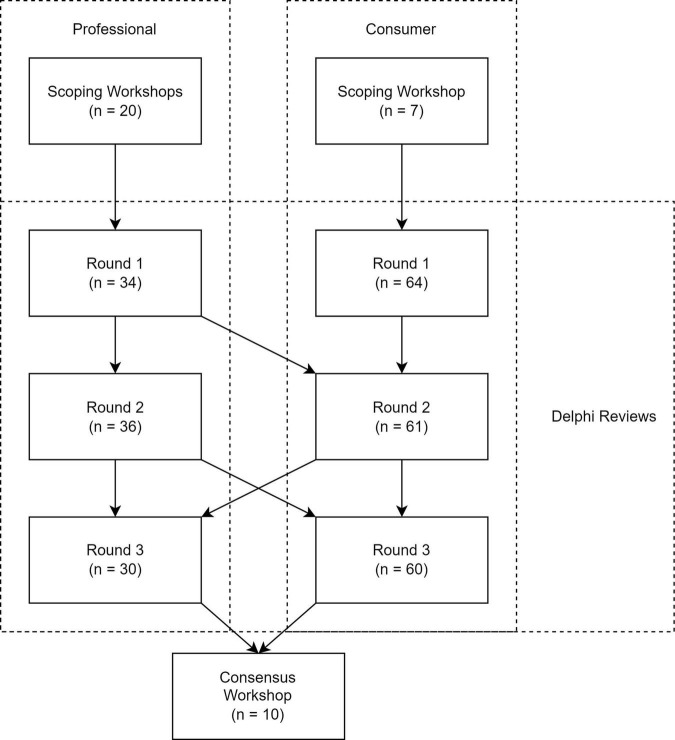
Overall structure of the research study, including the number of participants in each workshop and Delphi round. Information that was used to develop subsequent stages is indicated by arrows.

The overall structure of this research study is shown in Figure 1. Two groups of participants took part in this study: (i) Professionals, and (ii) Consumers. A scoping workshop and Delphi review was conducted with each group, and a final consensus workshop was conducted with representatives of both groups. The Delphi reviews covered six sections: *Outcome Domains, Time of Collection*, *Methods of Collection, Parties Responsible for Collection, Reason for Collection*, and *National* O*utcomes Database*. Where information from a previous stage was used to inform or develop a subsequent stage, this is denoted by an arrow.

### Method

#### Scoping Workshops

Four in-person scoping workshops were used to establish the initial statements for the *Outcome Domains* section of the Delphi reviews. Three workshops were conducted with the Professionals group, one each in Sydney, Melbourne, and Brisbane. One workshop was conducted in Sydney with Consumers from around Australia, with travel costs covered by the research team.

At these workshops, structured brainstorming exercises were used to assist participants to identify a comprehensive long list of domains in which outcomes of hearing rehabilitation might be observed.

For the workshops conducted with the Professional group, the New South Wales Government Human Services Outcomes Framework was used to guide brainstorming (Routledge, 2017). This framework identifies seven broad areas in which outcomes of health and human services interventions might be observed: Education and Skills, Economic, Health, Home, Safety, Empowerment, and Social and Community.

For the workshop conducted with the Consumer group, participants were asked to define “personas” for people involved in hearing services, including people with hearing loss, their family members, clinicians, and policymakers. For each of these personas, participants then brainstormed markers of successful hearing rehabilitation (e.g., “My husband and I doing more things together”) and markers of unsuccessful hearing rehabilitation (e.g., “Me getting frustrated by having to repeat things”).

The lists of identified outcome domains from the workshops were then combined by the research team, and duplicates were removed.

#### Delphi Reviews

A Delphi review is an iterative process in which respondents are asked to complete a series of surveys (rounds), with subsequent rounds including summary information about the responses to the previous round, allowing participants to re-evaluate their previous rating of a statement based on any emerging group consensus (Helmer, 1967). The Delphi technique is useful for building consensus among experts with regard to their areas of expertise (Hsu and Sandford, 2007), and has been used successfully for the development of clinical guidelines and rehabilitation approaches in hearing healthcare (Barker et al., 2015b; Sereda et al., 2015; Ferguson et al., 2018). In the present Delphi Review, each round was conducted *via* an online survey.

Two Delphi reviews, each of three rounds, were conducted with the Professional and Consumer groups separately. These reviews utilised different surveys and slightly different questions, targeted to the two different populations. Where the same question was asked of both groups, summary information from both groups was presented, allowing respondents to use information from both groups of stakeholders in their re-evaluation (see Figure 1).

The Delphi reviews covered six sections. Three of these (*Outcome Domains*, *Methods of Collection*, and *National Outcomes Database*) were asked of both groups, and three (*Time of Collection*, *Parties Responsible for Collection*, and *Reason for Collection*) were asked only of the Professionals group, as it was felt by the research team that these would likely be out of the scope of understanding of non-professionals.

For both groups, the standard *agreement* rating item was a five-point Likert item with anchors Strongly agree, Agree, Neither agree nor disagree, Disagree, and Strongly disagree. The standard *importance* rating item was a five-point Likert item with anchors Very important, Important, Neither important nor unimportant, Unimportant, and Very unimportant. The standard *comfortableness* rating item was a five-point Likert item with anchors Very comfortable, Comfortable, Moderately comfortable, Slightly comfortable, and Not at all comfortable. The relevant rating item was chosen for congruence with the statements presented to participants; a full list of statements as presented is available in Supplementary Material.

Consensus throughout the Delphi reviews was defined *a priori* as a consensus percentage of 80% or greater for each item, that is the proportion of respondents rating a statement as follows:

• for agreement:
  ∘ Agree or Strongly Agree, or
  ∘ Disagree or Strongly disagree;
• for importance:
  ∘ Very important or Important, or
  ∘ Unimportant or Very unimportant.

Comfortableness was not used to determine consensus, but only to determine consensus rankings.

To allow for discrimination between items beyond whether they reached consensus or not, consensus rankings were calculated for some items, determined using the Kemeny-Young method (Kemeny, 1959). This method, which has to our knowledge not been used previously in hearing research, generates the consensus ranking(s) of items that have the largest number of total pairwise agreements with the rankings provided by participants. The number of pairwise agreements is calculated by taking every possible pair of items, determining which item is ranked higher in the potential consensus ranking, and then counting the number of participant-provided rankings that also rank that item higher. In some cases, such as where all respondents ranked items in the same order, this ranking is unique, although in some cases the method produces “ties,” where there is insufficient information in the data to be able to definitively place one item above another. This method was applied by treating each person’s responses as a single ranking, in which those items rated as Strongly Agree (or Very important, or Very comfortable) are ranked above those items rated as Agree (or Important, or Comfortable), and so on.

For example, consider a set of three items which have been ranked by participants. In this case, there are 13 possible orderings to consider as the consensus ranking. With the set of responses described in Table 1, the potential consensus ranking Item 1 > Item 2 > Item 3 has three pairwise agreements with Person A (Item 1 > Item 2, Item 2 > Item 3, and Item 1 > Item 3), two agreements with Person B (Item 1 > Item 2 and Item 1 > Item 3), and two agreements with Person C (Item 1 > Item 3 and Item 2 > Item 3) for a total of seven pairwise agreements. If this process is repeated for all 13 possible orderings (13 = 8 total orderings plus 5 combinations involving ties), it can be seen that this ranking (Item 1 > Item 2 > Item 3) has the highest total and is therefore the Kemeny-Young consensus ranking.

**TABLE 1:** Synthesised responses to illustrate Kemeny-Young method.

	Person A	Person B	Person C
Item 1	Strongly agree	Agree	Agree
Item 2	Agree	Disagree	Agree
Item 3	Disagree	Neither agree nor disagree	Disagree
	Item 1 > Item 2 > Item 3	Item 1 > Item 3 > Item 2	(Item 1 = Item 2) > Item 3
K-Y ranking Item 1 > Item 2 > Item 3 Total of seven pairwise agreements	Three agreements (Item 1 > Item 2; Item 2 > Item 3; Item 1 > Item 3)	Two agreements (Item 1 > Item 2; Item 1 > Item 3)	Two agreements (Item 1 > Item 3; Item 2 > Item 3)

Determination of a consensus ranking is one of the most difficult problems to solve computationally, as the number of possible consensus rankings (possibly with ties) that need to be checked grows superfactorially (due to ties) with the number of items to be ranked (Bartholdi et al., 1989; Biedl et al., 2006): while there are 13 possible orderings for three items, there are 75 for four items, 541 for five items, approximately 102 million for 10 items, and over 230 trillion for only 15 items. As a result, as the number of items that have been ranked increases, it becomes computationally not feasible to determine the optimal consensus ranking definitively, and heuristic methods that provide computational approximations to the optimal ranking are required. In this study, where small numbers of items were to be ranked (<15), the branch and bound algorithm (which definitively determines the optimal consensus ranking) could be computed in a reasonable time and was used (<4 h; Emond and Mason, 2002). Where a larger number of items were to be ranked, the fast computational approximation developed by Amodio et al. (2016) (stylised “FAST”) was used instead.

#### Consensus Workshop

A summary of results from the *Outcome Domains*, *Time of Collection*, and *Methods of Collection* sections of the Delphi Reviews was distributed to workshop participants prior to the workshop.

At the online workshop, the top five outcome domains as determined by the Consumer group and the top five outcome domains as determined by the Professional group were discussed. Due to similarities between outcome domains across the groups, seven were identified as separate constructs. These were then presented to the workshop and discussed in detail, to ensure that participants had a coherent shared understanding of each domain.

Participants then separated into two groups to discuss the domains and their importance. Project team staff attended these groups but did not participate in the discussion beyond answering questions about the methodology and the results.

Participants then individually and anonymously ranked the domains from most important to least important. The summary of the individual rankings was presented to the group and discussed until unanimous agreement was reached on a short list of domains that should be recommended for collection from all people receiving hearing services in Australia.

Some discussion was also had regarding methods and time of collection of outcome measures, which was synthesised qualitatively and is summarised below.

#### Final Recommendations

The research team met to synthesise the results from all phases of the work, primarily the Consensus Workshop and literature review of potentially applicable outcome measures, into specific interim recommendations for the assessment of hearing rehabilitation in clinical and research practice. Discussion continued until unanimous agreement was reached.

### Participants

#### Professionals

This group comprised Professionals involved in the hearing industry in Australia. Potential participants were identified by the research team by brainstorming within each of the categories of hearing researchers, representatives of professional organisations, hearing service organisations, industry organisations such as hearing device manufacturers, and hearing consumer advocacy organisations. This resulted in a list of 59 people who were invited to take part in the study, with 43 (73%) consenting to take part.

Participants were invited to join in one of three initial scoping workshops, which were conducted in Brisbane (*n* = 6), Melbourne (*n* = 7), and Sydney (*n* = 7). Following these scoping workshops, several other potential participants were identified by workshop participants as being people who would be interested in contributing to the Delphi review, and were added to the list, giving a total of 79 potential participants for the Delphi review, of whom 50 (63%) completed at least one round of the Delphi review. Of these, 19 (38%) completed only one round, 16 (32%) completed only two rounds, and 15 (30%) completed all three. Participation in a future round was not contingent on completion of all previous rounds.

All categories used to identify potential participants were represented in the scoping workshops.

#### Consumers

Four hearing advocacy organisations active in Australia were identified (Better Hearing Australia Brisbane, Deafness Forum, Hearing Matters Australia, and Soundfair) and invited to nominate one or more representatives who identified as people with hearing loss. Seven representatives across all these groups took part in the initial scoping workshop.

Following the workshop, the organisations were invited to share a link to the first round of the Delphi review with their members, and all did so. Participants who responded to round 1 of the Delphi Review (*n* = 64) were then invited to participate in rounds 2 and 3. Of these, 55 (86%) completed all three rounds, 6 (9%) completed only two rounds, and 3 (5%) completed only round one. There was higher engagement with the review process in this group that in the Professional group, potentially due to the self-selected nature of the participants.

#### Final Consensus Group

A group of potential participants (*n* = 18) was selected by the research team from those in the Professional group who had responded to at least 2 rounds of the Delphi Review (*n* = 32). In the selection of these potential participants, the categories used at the potential participant identification stage were considered to ensure broad coverage of the Australian hearing industry. One potential participant from each consumer organisation was then added. The resulting 22 potential participants were then invited to take part in an online workshop (due to COVID-19), with 10 attending.

### Material

The Delphi Reviews were conducted using three rounds of electronic surveys. A summary of the types of responses invited (e.g., ranking of importance) for each section of each survey can be seen in Table 2.

**TABLE 2:** Types of response invited for each section of the Delphi review surveys.

Section	Round 1	Round 2	Round 3
Outcome domains	Professional: Rating of importance and open-ended question	Professional: Rating of importance	Professional: Rating of importance and ranking of importance
	Consumer: Rating of importance and open-ended question	Consumer: Rating of importance	Consumer: Ranking of importance
Methods of collection	Professional: Open-ended questions	Professional: Ranking of importance	Professional: Ranking of importance
		Consumer: Rating of comfortableness	Consumer: Ranking of importance
National Outcomes Database: Purposes and drawbacks	Professional: Open-ended questions	Professional: Rating of importance	Professional: Rating of importance
		Consumer: Rating of importance.	Consumer: Rating of importance
National Outcomes Database: Potential stakeholders to run a database		Consumer: Rating of comfortableness.	
Time of collection: Time points	Professional: Open-ended question	Professional: Ranking of importance	
Time of collection: Benefits and drawbacks of time points	Professional: Open-ended question	Professional: Rating of agreement	Professional: Rating of agreement
Reasons for collection	Professional: Open-ended question	Professional: Rating of agreement	Professional: Rating of agreement
Parties responsible for collection	Professional: Open-ended question	Professional: Rating of agreement	Professional: Rating of agreement

In round 1, participants were asked open-ended questions about the topics, which were then synthesised into statements for rating in subsequent rounds.

In rounds 2 and 3, statements were presented for rating using one of the standard items or ranking, along with summary information about responses to any previous rounds of ratings or rankings. In the *Methods of Collection* and *National Outcomes Database* sections, questions asked were similar between the two groups, and so summary information for both groups was presented.

In the *Outcome Domains* section, statements were rated using the standard importance item. As statements had already been determined during the scoping workshops, in Round 1 they were presented for rating, along with an open-ended question to allow participants to add any outcome domains that they felt were missing. In Round 3 participants were also asked to select, in ranked order, the top five domains, which were used to generate a consensus ranking. Due to the large number of outcome domains that reached consensus in Round 1 among Consumer participants, in Round 2 Consumers were presented with only the top 10 outcome domains from the previous round (as determined by the Kemeny-Young method), as well as five additional domains synthesised from open-ended responses. All of these reached consensus as being important, and so in Round 3 Consumer participants were not asked to rate, but only to rank the domains.

In the *Time of Collection* section, Professional participants were asked about the different time points at which outcomes could be collected, and why. Responses were synthesised into four major time points and a set of statements regarding the potential reasons why these time points might be useful. In Rounds 2 and 3, Professional participants were asked to rank the four time points in order of importance, and to rate their agreement with the statements using the standard *agreement* rating item. The ranked time points were used to generate a consensus ranking.

In the *Methods of Collection* section, Professional participants were asked about potential methods that could be used to collect outcomes, and the benefits and drawbacks that might be associated with each method. Professional participants were asked to rank the top five most important methods of collection in both Rounds 2 and 3 to facilitate the recommendation of a single method of collection at the close of the study. Consumer respondents were asked to rate the *comfortableness* of each method in Round 2, and to rank the top five most important methods in Round 3.

In the *Parties responsible for collection* section, Professional participants were asked about different parties (e.g., clinicians, a Government agency, or GPs) who could be responsible for the collection of outcome measures in the Hearing Services Program, and the potential benefits and drawbacks of each these parties undertaking outcomes collection. Statements were rated by Professional participants using the standard *agreement* rating item in subsequent rounds.

In the *Reasons for Collection* section, Professional participants were asked about reasons why different stakeholders might find it important for outcomes to be measured. Statements were rated by Professional participants using the standard *agreement* rating item in subsequent rounds.

In the *National Outcomes Database* section, Professional participants were asked about the potential beneficiaries, benefits, and drawbacks of the development of a national database of outcomes for hearing rehabilitation. The beneficiaries and benefits were synthesised into potential purposes for such a system. The potential purposes and drawbacks were then presented to both groups, and participants were asked to rate them using the standard *importance* rating item. In Round 2, Consumer participants were also asked how *comfortable* they would feel with a range of different stakeholders running a national outcomes database.

Questions asked in each section are available as Supplementary Material.

## Results

### Delphi Reviews

Results for this section are shown in Tables 3–8. In each table, items are ordered by the consensus ranking, when both consensus ranking and percentage are available, and then by the consensus percentage when ranking is tied (ordered from unanimous agreement to unanimous disagreement). Consensus percentages meeting the predefined criterion (80%) are shown in bold. In some cases, there is disagreement between the ordering implied by the consensus percentages and that obtained using the consensus ranking procedure, as the consensus percentage method treats “agreement” from one participant and “strong agreement” from another as equivalent. As the ranking takes individual preferences between domains into account, ranking should be considered a more accurate measure of consensus preference. However, as the ranking of an item is dependent on preferences for other items in the set, no strict criterion for ranking can be applied.

**TABLE 3:** Results from the outcome domains section among the professional group.

Domain	Consensus percentage	Consensus ranking
Improved communication ability	**100%**	1
Improved communication in groups	**97%**	2
Improved personal relationships	**100%**	3
Improved self-management ability	**87%**	4
Improved well-being	**87%**	5
Improved participation in activities	**97%**	6
Improved social engagement	**90%**	7
Increased use of hearing aids	77%	8
Improved sense of empowerment	**80%**	9
Increased independence	**87%**	10
Reduced social isolation	**97%**	11
Reduced loneliness	**83%**	12
Reduced listening effort	**97%**	13
Improved community engagement	**83%**	=14
Improved access to education	53%	=14

*Consensus percentages meeting the consensus criterion are shown in bold.*

**TABLE 4:** Results from the outcome domains section among the consumer group.

Domain	Consensus percentage	Consensus ranking
I can live my life independently	**90%**	1
I can communicate well with my family	**100%**	2
I can communicate effectively with people	**98%**	3
I am able to do the things that I want to do	**95%**	4
I hear clearly with my hearing aids	**95%**	5
I can use my hearing aids effectively	**100%**	6
My hearing impacts less on my family	**98%**	7
I have the skills I need to communicate	**93%**	8
I have more control over my hearing	**88%**	9
I trust my hearing care professional	**93%**	10
My hearing aids are comfortable	**95%**	11
I can use the telephone effectively	**88%**	12
I am better able to hear the TV as a result of my hearing care	**84%**	13
I am able to participate in the social events that I want	**90%**	14
I am satisfied with the hearing care I receive	**95%**	15

*Consensus percentages meeting the consensus criterion are shown in bold.*

**TABLE 5:** Statements from the time of collection section among the professional group.

Statement	Consensus percentage
A baseline measure should be obtained at or prior to fitting of a device to help determine the course of treatment intervention	**93%**
A baseline measure should be obtained at or prior to fitting of a device to assess future progress	**93%**
The final outcome measure should not be collected any sooner than 3 months as clients may not have acclimatised to their devices	**83%**
Outcome measures should be obtained multiple times during a year to assess the course of the rehabilitation intervention	77%
An outcome measure should be obtained at around the 3-months period, as clients struggle with device compliance around this period	50%
Outcome measures are likely to capture a more holistic view if conducted 12-months post fitting	50%
Outcome measures are likely to capture a more holistic view if conducted 6-months post fitting	47%

*Consensus percentages meeting the consensus criterion are shown in bold.*

**TABLE 6:** Rankings of methods of outcomes collection.

Statement	Professionals consensus ranking	Consumers consensus ranking
The hearing care professional fills out a questionnaire with the client face to face	1	1
The client fills out a paper questionnaire that is posted to them by their hearing care professional	2	=3
The client fills out a questionnaire (paper or electronic) with their GP	3	=3
The client fills out a paper questionnaire that is posted to them by their GP	4	=3
The client fills out a paper questionnaire (or electronically on a tablet) and returns it to their hearing care professional or the receptionist	5	=3
The hearing care professional fills out a questionnaire with the client over the telephone	6	=3
The client fills out an online questionnaire that is emailed to them by their hearing care professional	7	2

**TABLE 7:** Statements from the parties responsible for collection section among the professional group.

Statement	Consensus percentage	Direction
Outcomes are best collected by the client’s own hearing care professional because outstanding problems experienced by the client can be responded to more readily	60%	Agree
Outcome should be collected by a third party independent of the hearing care organisation to avoid the potential for bias	50%	Agree
Clients will be more honest if outcomes are collected by someone independent of their hearing care organisation	43%	Agree
Outcomes are best collected by the client’s own hearing care professional because the client is familiar with the hearing care professional and they are familiar with the client	33%	Agree
Outcomes should be collected by hearing advocacy groups because they are less likely to show any bias	33%	Disagree
Outcomes should be collected by hearing care professionals because they are less likely to show any bias	37%	Disagree
A Government body, e.g., the Hearing Services Program is the best placed group to collect outcomes	50%	Disagree

*For each statement, the consensus percentage is given, along with the direction in which that consensus percentage was calculated. For example, for the first item, 60% of people rated the item as Agree or Strongly Agree, and a smaller percentage rated it as either Disagree or Strongly Disagree.*

**TABLE 8:** Statements from the reasons for collection section among the professional group.

Statement	Consensus percentage
To provide an evidence base to help inform clinical decision-making	**97%**
To inform hearing care professionals as to the need for further interventions for their clients	**97%**
To ensure that services offered are providing benefit to clients	**97%**
To ensure that hearing care professionals are providing appropriate hearing care services to their clients	**94%**
To provide a benchmark against which clinical services can be measured	**94%**
To demonstrate whether the Voucher Scheme is positively impacting clients	**94%**
To demonstrate the success of the rehabilitation programme for the client	**94%**
To enable hearing care organisations to monitor consistency of practice	**90%**
To help inform the client’s rehabilitation journey and management plan	**87%**
To provide evidence for the effective use of government resources	**84%**
To help promote a more holistic approach to hearing rehabilitation rather than focus solely on hearing aids	**84%**
To enable the hearing care professional to compare management approaches, e.g., when trying a different rehabilitation option	**84%**
To help the Government and other funders target poorly performing hearing care organisations for auditing	**81%**
To facilitate the identification of hearing care professionals within an organisation who require more training or assistance	77%
To provide population data to health researchers	74%

*Consensus percentages meeting the consensus criterion are shown in bold.*

#### Outcome Domains

The primary question for this section was “What outcome domains should be measured as markers of success of hearing rehabilitation?”

Results from the Professional group are shown in Table 3. For each item, the consensus percentage and the consensus ranking from the final ranking task are shown. The consensus criterion was met for 13 domains and not for two domains.

Most domains that were identified through this process were psychosocial. Notably, the consensus criterion was not reached for the domain “Increased use of hearing aids.”

Results from the Consumer group are shown in Table 4. There was consensus beyond the predefined criterion on every domain presented. As there were several domains that were excluded after Round 1 and not included for Rounds 2 and 3, it is possible that a subset of these, should they have been presented, may also have reached the predefined criterion for consensus.

#### Time of Collection

The primary question for this section was “At what time point(s) should outcome measures be collected, and why?” It was conducted in two parts: by presenting respondents with four specific time points for ranking, and then by presenting a set of statements.

The four specific post-fitting time points identified were, in ranked order from most to least preferred, at 3 months following the fitting, at 6 months following the fitting, at 12 months following the fitting, and at the follow-up appointment (commonly conducted between one and 3 weeks post-fitting in Australia).

The statements and consensus percentages for this section are shown in Table 5. The highest consensus percentage at 93% related to the two statements on the use of baseline measures prior to device fitting, which was strongly supported by respondents. There was consensus reaching the criterion for three statements and not for four statements.

#### Methods of Collection

The primary question for this section was “What different methods could be used to collect outcome measures?”

Consensus rankings for this section are shown in Table 6. Note that the consensus ranking method was unable to distinguish between statements ranked third among respondents in the Consumers group for five of the statements. The orderings obtained from Professional group and from the Consumer group were notably different, with the second preference among Consumers (an online questionnaire emailed by the hearing care professional) being ranked last by the Professional respondent group.

#### Parties Responsible for Collection

The primary question for this section was “Thinking of patients being seen for hearing rehabilitation, who could potentially collect outcome measures?”

Statements and consensus percentages for this section are shown in Table 7. The predefined consensus criterion was not reached for any of the statements. As the results tended toward disagreement for some of the statements, whether the consensus percentage relates to agreement or disagreement is also shown in the table. It should be noted that as “Neither agree nor disagree” was a valid option for respondents, consensus percentage for agreement and consensus percentage for disagreement do not sum to 100%. For example, 33% consensus toward agreement displayed in the table indicates that fewer than 33% of respondents responded “Disagree” or “Strongly disagree,” with the remainder responding “Neither agree nor disagree.”

#### Reasons for Collection

The primary question for this section was “Why might it be important to clinicians providing hearing services/hearing service providers/Government that outcomes are measured?”

The statements and consensus percentages are shown in Table 8. Consensus reached the pre-defined criterion on 13 statements and did not reach the criterion on two statements. A broad array of potential reasons for collecting outcome measures with differing beneficiaries was identified, including hearing care professionals, Government, hearing care organisations, and the public.

#### National Outcomes Database

The primary questions for this section were: “Are there any people who you think might benefit from a national outcomes database? What are the potential benefits of a national outcomes database? What are the potential drawbacks associated with having a national outcomes database?” Detailed results of this section, including consensus percentages and rankings, are available in Supplementary Material, and a summary is provided below.

Both groups agreed that a database should be designed to promote person-centred care, to help determine best practice, and to provide a national standard for hearing care. Both groups also agreed that such a database should be designed to measure the impacts of hearing loss beyond the person themselves, on their partners, family members, and friends. The Consumer group, but not the Professional group, agreed that a database should support clients to choose hearing services and providers and help identify poorly performing clinicians and services.

Both groups were agreed that the accuracy and relevance of measures and the integrity of the data was highly important, with misuse of data by professionals or organisations a significant concern. Professional participants were also concerned with the potential for data breaches and use of the data to justify funding cuts.

Consumer participants felt more comfortable with organisations that might be considered independent from both the hearing industry and Government running a national outcomes database, including independent research organisations, professional associations, and universities.

### Consensus Workshop

The synthesised domains as presented to the consensus workshop are available in Supplementary Material.

During initial discussion to ensure that participants understood the domains as presented, participants decided that “Improved participation in activities” should instead refer to reduction of “participation restrictions,” as it was felt that it was unreasonable to expect that the provision of hearing rehabilitation alone would result in increased participation by patients in social activities. Rather, participants felt that while hearing rehabilitation could reduce the barriers to participation caused by the hearing loss, the social and psychological effects of long-standing hearing difficulty may result in some continued persistence of patterns of reduced participation for a period of time following any reduction in participation restrictions.

Following this discussion, participants separated into groups to further discuss the domains, and anonymously ranked the domains individually. A summary of the results of individual prioritisation for both groups are shown in Table 9.

**TABLE 9:** Summary of rankings of individual domains provided by participants in the consensus workshop.

Domain	1^st^	2^nd^	3^rd^	4^th^	5^th^	6^th^	7^th^
Improved communication ability	10	0	1	0	0	0	0
Improved personal relationships	1	4	5	1	0	0	0
Improved well-being	0	5	0	3	0	0	1
Reduced participation restrictions	0	1	1	5	3	1	0
Increased independence	0	1	1	1	7	1	0
Improved perception of clarity	0	0	1	0	1	3	6
Improved self-management ability	0	0	0	1	0	6	4

*For each individual domain presented to participants, the table shows the number of participants ranking it first, second, etc. This is the same format in which these data were made available to participants.*

After prioritisation, group discussion was undertaken to identify which outcome domains were considered core for assessment of hearing services. Discussion focussed on the importance of capturing the full breadth of outcomes experienced by patients, the importance of domains that were clearly articulable and comprehensible by patients and clinicians, and on selecting domains that would be directly modifiable by rehabilitation efforts.

Following the discussion, the group decided unanimously that four outcome domains should be recommended as part of a CODS for self-report in hearing rehabilitation in Australia. These were, in order of importance: (1) communication ability, (2) personal relationships, (3) well-being, and (4) participation restrictions. The group stressed that all seven domains presented to the group were important and should be considered for settings where the collection of additional outcomes is possible.

Following the discussion of which outcome domains should be measured following hearing rehabilitation, additional discussion was had regarding the time points at which outcomes should be measured, and which outcome measures should be used. There was no decision made regarding a positive answer to either question, with participants agreeing that these questions should be answered with reference to the research literature. Participants felt that the time of collection may differ between particular outcome measures and should therefore be determined with reference to research relevant to each particular outcome measure.

### Final Recommendations

Three overarching recommendations were made as a primary output of this project. It should be noted that these recommendations were made specifically for the Australian publicly funded hearing rehabilitation context, and with the assumption that outcomes would be collected to facilitate their tracking and improvement over time.

1) Target the outcome domains “communication ability,” “personal relationships,” “well-being,” and “participation restrictions” when assessing hearing rehabilitation.
2) Measure within these domains at baseline and then following the conclusion of the rehabilitation, with a delay of at least 3 months being recommended.
3) Establish an independent body to develop a standardised outcomes instrument and mechanism for outcomes collection.

The detailed recommendations from this project are available in Supplementary Material.

## Discussion

### Outcome Domains

This study identified four primary outcome domains that are recommended as part of a core outcome domain set for the self-report evaluation of individual hearing rehabilitation programmes: communication ability, personal relationships, well-being, and participation restrictions.

Many currently available outcome measures used as measures of the success of hearing rehabilitation focus on improvements in communication ability, including the Glasgow Hearing Aid Benefit Profile (GHABP; Gatehouse, 1999), the Abbreviated Profile of Hearing Aid Benefit (APHAB; Cox and Alexander, 1995), and the International Outcomes Inventory for Hearing Aids (IOI-HA; Cox et al., 2000). The delineation of specific subdomains of communication ability in this study (communication with family and communication in groups) suggests that in addition to generalised measures of patients’ overall communication ability in their everyday lives, specific measures or subscales highlighting difficulties or successes in these identified areas are also required for a comprehensive assessment of communication ability. Many commonly used measures for the assessment of communication ability validated in hearing rehabilitation do not address these subdomains separably, although some measures include these aspects, such as the SOS-HEAR (Scarinci et al., 2012), the Self-Assessment of Communication (SAC; Schow and Nerbonne, 1982), and the GHABP. A notable exception is the Communication Performance subscale of the Communication Profile for the Hearing Impaired (CPHI; Demorest and Erdman, 1987), which includes several items related to communication both in group social situations and with family around the home. However, these items do not form separable subscales, making it difficult to assess communication on the subdomains in a separable way (Demorest and Erdman, 1986). The instrument also includes items that may be inappropriate for some people, such as hearing in lectures and religious services. Further work may be required to develop an instrument that can assess communication ability both across life as a while and in particularly meaningful situations for a wide variety of people.

The most specific measure of hearing-related participation restriction currently available, the Social Participation Restrictions Questionnaire (SPaRQ), focuses primarily on social participation (Heffernan et al., 2018a,b). Participation restrictions due to hearing loss manifests across a range of kinds of non-social participation, including in employment, education, domestic settings, and political life (Danermark et al., 2013; Granberg et al., 2014c), and so additional measures or expansion of the SPaRQ is likely to be necessary to enable comprehensive assessment.

There currently are no measures validated in hearing rehabilitation for the assessment of personal relationships or general well-being, although general measures of well-being such as the Warwick-Edinburg Mental Well-Being Scale (WEMWBS) are available (Tennant et al., 2007). Application of modern psychometric methods to the WEMWBS has been useful in the derivation of short-form measures with desirable measurement characteristics, suggesting that item inventories of this kind may prove useful as a starting point for the development of shorter, more specific measures of well-being benefit following hearing rehabilitation (Houghton et al., 2017). There is also ongoing work exploring the nature of hearing-specific well-being (Vercammen et al., 2020; Humes, 2021), which may result in measures of well-being that are more sensitive to hearing rehabilitation, although care should be taken when selecting these instruments to ensure that the breadth of well-being as an outcome domain—that it includes all aspects of life, not just hearing—is not lost.

### Collection Considerations

With respect to the time points at which outcomes should be collected, there was disagreement between professional stakeholders in the Australian hearing industry, reflecting the lack of research into the optimal time to collect outcomes measurements. While there is good evidence that auditory ability stabilises quickly after hearing aid fitting (Dawes et al., 2014b), the period following fitting, particularly for new users, is marked by ongoing adjustment during which results obtained from outcome measures may be expected to change (Turner et al., 1996). There are also a variety of personal factors which may affect the rate at which a person adjusts to hearing aids (Dawes et al., 2014a), and it is unclear how those factors may affect different domains of adjustment. As a result, further research will be required to establish the optimal time post-fitting for any particular outcome measure to be applied.

While both Consumer and Professional groups agreed that outcomes could be appropriately collected by hearing care professionals face-to-face with a patient, the difference in preference for online delivery of questionnaires—Consumer respondents ranking it second only to face-to-face collection by a hearing care professional while Professional respondents ranking it as least preferred—suggest that a range of methods are likely to be useful in practice. The principles of experience-based co-design suggest that health services, policymakers, and consumers should be involved in the design of services and the selection of appropriate metrics for their assessment (Donetto et al., 2014), and the results of this study suggest that the methods of collection of those metrics may also be a valuable subject of co-design methods. Further work canvasing views of outcomes collection methods may also identify groups of consumers and service staff who benefit from varying methods of outcome measurement collection, requiring a multi-method approach to implementation into hearing health services.

There was substantial overlap in the identified reasons for collecting outcomes and the purposes of establishing a national database of patient outcomes. Both Consumer and Professional groups highlighted the importance of promoting patient-centred care beyond solely the provision of hearing aids, the comparison of professional practice and outcomes to national benchmarks, the value of outcomes to Government and other funders in developing policy, and potential enhancements in the development of evidence-based hearing care. This overlap suggests that to the participants in this study these two ideas—that outcome measurements should be collected and that outcome measurements should be combined and analysed across health systems—may be conceptually inseparable. Indeed, several of the reasons identified for collecting outcomes, such as the targeting by Government of auditing activities, are likely to only be possible through a centralised outcomes storage and analysis system.

Respondents were clear that centralised outcomes collection systems should be designed to maximise their benefits for various parts of the hearing health system, including hearing healthcare organisations and professionals, other healthcare providers, the public, and policymakers. When designing and implementing these systems, a broad base of stakeholders should be involved in the design and implementation of data products, ensuring their applicability across the health system. Consumers, perhaps unsurprisingly, felt that such a system could and should provide important information to consumers to support their hearing rehabilitation decision-making. The provision of information to consumers alone, however, is not sufficient to ensure that they can use that information to support healthcare decision-making; careful design of the consumer-accessible outputs of these systems will be necessary to ensure that outcomes information can be useful to consumers (Hibbard and Peters, 2003).

In addition to the benefits of aggregating outcome measurements across patients for services and systems evaluation, the results of the present study also highlight the immediate utility of outcome measurements to clinicians as a basis for decisions about the future progress of individual rehabilitation programmes. Making patient outcome measurements available to clinicians and health services may therefore provide an immediate and direct benefit to the care of the patient whose outcomes are being measured. In addition to the use of baseline measurements to support rehabilitation programme planning (such as the determination that a patient may be more likely to benefit from intensive communication training to address difficulties in particular situations, or from a referral to psychological or social support to ameliorate the effects of long-standing participation restriction), outcome measurements may identify patients whose progress has been less than might be expected, prompting additional intervention from the clinician. Providing those results that are available to hearing care professionals and organisations to the patient, both in aggregate and individualised format, may provide significant benefits to clinical practice, improve engagement and uptake by hearing care professionals and service delivery organisations, and smooth the implementation of outcome measurement within hearing health systems.

Concerns relating to the development of a national outcomes database largely related to the validity, quality, trustworthiness, and comprehensiveness of the data stored within it. Both groups expressed concerns that there could be significant potential for interested parties (particularly hearing care professionals or organisations) to modify or misrepresent data in an attempt to appear more favourably in any aggregate results. This concern has implications for the ways in which data may be collected, as methods of data collection that directly involve hearing care professionals or organisations may be viewed as more susceptible to misuse than those that bypass hearing services entirely.

Interestingly, the potential impacts on professionals and organisations of receiving poor outcomes results were not considered important by either Consumer or Professional participants. Publication of health quality data can prompt quality improvement within health systems (Fung et al., 2008), with systematic and structured outcomes an important support to quality improvement activities (Kampstra et al., 2018). Within hearing rehabilitation, improvements in service quality have been seen following the publication of outcomes data in the ongoing quality register of hearing rehabilitation clinics in Sweden (Nationellt Kvalitetsregister Hörselrehabilitering, 2019). This suggests that the ongoing collection and publication of client-centred outcomes may support a move toward improving the quality of hearing rehabilitation.

### Limitations

While the results of the present study do provide important guidance for researchers, clinicians, and policymakers in the selection of outcome domains for the assessment of hearing rehabilitation, the decision in the early stages of the Delphi Review conducted with the Consumer group to restrict to a manageable number of outcome domains does mean that this list should not be considered a comprehensive description of the areas in which consumers of hearing services might experience or seek benefit from hearing rehabilitation. Indeed, several items that reached consensus in the Professional group, including reductions in social isolation and the ability to communicate in groups, were filtered out of consideration by Consumer participants at this stage. In addition, a number of items that might be considered valuable by researchers or clinicians—including feelings of empowerment, improved access to paid and volunteer work, and confidence in the effectiveness of hearing services—were also excluded from further consideration by Consumer participants. As a result, this work does not preclude the usefulness of other domains that have not been listed above, or of measures that assess other aspects of benefit. For example, where a comprehensive assessment of benefit of hearing rehabilitation is desired, the use of a general measure of improvement such as the Clinical Global Impression, which has been adapted for use in hearing rehabilitation (Öberg et al., 2009), may capture a more holistic measure of benefit, supporting the use of these more specific measures. Finally, the Delphi method used in this study involved a self-selected group of participants, and its reliance on internet-delivered text may have posed a barrier to participation for culturally and linguistically diverse Australians, those with cognitive or other difficulties, or low access to technology. Further work is required to ensure that the domains identified are indeed appropriate for the assessment of all consumers of Government-funded hearing services in Australia.

### Strengths

This study includes consumers of hearing services as primary participants in the development of recommendations for the assessment of hearing rehabilitation, which has not previously been done in this field. In addition to providing a possible example to future researchers seeking to include consumers as domain experts in their research, we believe that direct involvement of consumers in research is vital to the principles of patient- and family-centred care. It is also the first in the hearing literature to make use of the Kemeny-Young method for consensus ranking, a data-driven method with useful properties including satisfaction of the Condorcet criterion (that is, it will correctly identify the choice that is preferred over every other choice by most raters should such a choice exist) and the ready availability of “off-the-shelf” algorithms for both its exact computation and heuristic approximation.

### Conclusions

The recommendations from this study define a minimum patient-centred core outcome domain set that should be considered for the assessment of hearing rehabilitation in research and clinical practice. However, there is still significant research required to establish a set of outcome measures suitable for the measurement of each of these outcome domains. The selection of measurement instruments to be associated with a COS is a multi-stage process that will require considerable additional work, particularly given the identified lack of appropriate, validated measures for the identified domains (Prinsen et al., 2016). As part of this process, the measurement properties of developed or identified measures will need to be assessed. Preferably, this should be undertaken using modern psychometric methods such as those utilising Item Response Theory (IRT), which are particularly useful when assessing psychosocial, needs-based aspects of health (Tennant et al., 2004).

In general, these results have identified, through a consensus approach, a core outcome domain set that might be considered for the self-report evaluation of hearing rehabilitation and provide important background information for the design of methods to implement them across hearing healthcare systems. A broader set of self-report outcome domains that researchers and clinicians may also choose to collect in their particular context has also been identified. Furthermore, other outcome areas in addition to self-report, such as behavioural (e.g., speech perception, cognition) and physiological (e.g., electrophysiology) tests, need to be considered before there is a full COS for auditory rehabilitation. For self-report, which was the focus of this study, the range of suggested outcome domains, potential purposes for outcomes collection, and potential concerns with the establishment of centralised national outcomes collection and analysis systems strongly suggest that ongoing stakeholder engagement will be vital for the operationalisation of these results into any hearing healthcare system. In addition, significant further research is required on any selected or developed outcomes measurement instruments to determine the optimal time of outcomes collection following hearing rehabilitation.

## Data Availability Statement

The original contributions presented in the study are included in the article/Supplementary Material, further inquiries can be directed to the corresponding author.

## Ethics Statement

The studies involving human participants were reviewed and approved by Hearing Australia Human Research Ethics Committee. The patients/participants provided their written informed consent to participate in this study.

## Author Contributions

MF designed the study. MF and DA designed the Delphi review with subject matter advice from LH. DA managed the data collection, analysed the data, and wrote the manuscript. All authors interpreted the data, reviewed the manuscript and provided critical revision, and approved the submitted version.

## Conflict of Interest

The authors declare that the research was conducted in the absence of any commercial or financial relationships that could be construed as a potential conflict of interest.

## Publisher’s Note

All claims expressed in this article are solely those of the authors and do not necessarily represent those of their affiliated organizations, or those of the publisher, the editors and the reviewers. Any product that may be evaluated in this article, or claim that may be made by its manufacturer, is not guaranteed or endorsed by the publisher.
