# Supplementary material for: Defining a Patient-Centred Core Outcome Domain Set for the Assessment of Hearing Rehabilitation With Clients and Professionals
Source: Front Neurosci. 2022 May 3;16:787607. doi: 10.3389/fnins.2022.787607 (PMC9110701; doi:10.3389/fnins.2022.787607)

# Supplementary Materials

Table 1

Questions asked in the Outcome Measures section of the two Delphi Reviews

|  | Professional | Consumer |
| --- | --- | --- |
| Round 1 | Below are some potential outcome domains of hearing rehabilitation. On a scale of 1 (Very unimportant) to 5 (Very important), how Important do you think it is that we measure each one?  Are there any additional outcomes that you believe would be valuable to measure? | For each statement, how important do you think it is to measure this as an indication that hearing care has been effective?  Are there any other things that you think should be measured as a sign that your hearing care has been effective that were not mentioned in the previous sections? |
| Round 2 | For each of the outcome domains listed below, please indicate the extent to which it is important to measure. | For each of the following statements, please indicate the extent to which you think the statement is important or not in indicating hearing care success. |
| Round 3 | Previously in this section, you were asked to rate how important you think each of a set of outcome domains is to measure. Again, for each of the outcome domains listed below, please indicate the extent to which you think it is important to measure.  If you could only select the 5 most important outcome domains from the list below, which would they be? Please select 1 for the most important outcome domain, 2 for the next most important, and so on up to 5. Select 5 options only. | If you could only select the 5 most important outcome areas from the list below, which would they be? Please select 1 for the most important outcome area, 2 for the next most important, and so on up to 5. Select 5 options only. |

Table 2

Questions asked in the Time of collection section of the Professional Delphi Review

|  | Professional |
| --- | --- |
| Round 1 | At what time point(s) should outcome measures be collected, and why?  What are the benefits and drawbacks associated with each of these collection times, and why? |
| Round 2 | There are many time points to measure outcomes. Please rank in the order of importance these time points in which to measure outcomes. Please select 1 for the most important method, 2 for the next most important, and so on up to 4.  With regard to the time points at which outcomes measures should be collected, please indicate the extent to which you agree or disagree with each statement. |
| Round 3 | Previously in this section you were asked about when outcomes should be collected. Please indicate the extent to which you agree or disagree with each statement. |

Table 3

Questions asked in the Methods of Collection section of the two Delphi Reviews

|  | Professional | Consumer |
| --- | --- | --- |
| Round 1 | What different methods could be used to collect outcome measures?  What are the benefits and drawbacks of the different methods of collection? |  |
| Round 2 | There are many methods that could be used to collect outcomes. If you could only select the 5 most important methods from the list below, which would they be? Please select 1 for the most important method, 2 for the next most important, and so on up to 5. Select 5 options only. | Below are several methods that might be used to collect outcomes after hearing care. For each one, how comfortable would you feel using it? |
| Round 3 | Previously in this section, you were asked to rank the top five methods by which outcomes should be collected. Below are the rankings generated from the Hearing Professional and Consumer stakeholder ratings. In each case, 1 is the most preferred method, 2 is the next-most preferred, and so on. After looking at the rankings, if you could only select the 5 most important methods from the list below, which would they be? Please select 1 for the most important method, 2 for the next most important, and so on up to 5. Select 5 options only. | Below are some methods that might be used to collect outcomes after hearing care. If you could only select the 5 most important methods from the list below, which would they be? Please select 1 for the most important method, 2 for the next most important and so on up to 5. |

Table 4

Questions asked in the Parties responsible for collection section of the Professional Delphi Review

|  | Professional |
| --- | --- |
| Round 1 | Thinking of patients/clients being seen for hearing rehabilitation, who could potentially collect outcome measures?  What are the benefits and drawbacks associated with each of these people/groups collecting outcome measures? |
| Round 2 | Who should collect outcome measures? Please indicate the extent to which you agree or disagree with each statement. |
| Round 3 | Previously in this section you were asked about who should collect outcome measures following hearing rehabilitation. Please indicate the extent to which you agree or disagree with each statement. |

Table 5

Questions asked in the Reasons for collection section of the Professional Delphi Review

|  | Professional |
| --- | --- |
| Round 1 | Why might it be important to clinicians providing hearing services that outcomes are measured?  Why might it be important to hearing service providers that outcomes are measured?  Why might it be important to Government that outcomes are measured? |
| Round 2 | Please indicate the extent to which you agree or disagree with each statement. |
| Round 3 | Previously in this section, you were asked to think about the reasons why standardised outcome measures should be collected. Please indicate the extent to which you agree or disagree with each statement. |

Table 6

Questions asked in the National Outcomes Database section of the two Delphi Reviews

|  | Professional | Consumer |
| --- | --- | --- |
| Round 1 | Are there any people who you think might benefit from a national outcomes database?  What are the potential benefits of a national outcomes database?  What are the potential drawbacks associated with having a national outcomes database? |  |
| Round 2 | A national outcomes database would have many potential purposes. Thinking about a national database of outcomes for people being seen under the Australian Government's Hearing Services Voucher Program, how important is each purpose listed below?  A national outcomes database also has some potential negative effects. How important do you think each negative effect listed below is to consider in the design of such a system? | How comfortable would you feel if information about your hearing loss, rehabilitation, and outcomes were held in a database run by the following organisations?  Do you have any comments on the possibility of a National Database?  A national outcomes database could have many potential purposes. Thinking about a national database of outcomes for people being seen under the Australian Government's Hearing Services Program, how important do you think each potential purpose listed below is?  A national outcomes database also has some potential negative effects. Thinking about a national database of outcomes for people being seen under the Australian Government's Hearing Services Program, how important do you think each potential negative effect is to consider in the design of such a system? |
| Round 3 | A national outcomes database would have many potential purposes. Thinking about a national database of outcomes for people being seen under the Australian Government's Hearing Services Voucher Program, how important is each purpose listed below?  A national outcomes database also has some potential negative effects. How important do you think each negative effect listed below is to consider in the design of such a system? | A national outcomes database would have many potential purposes. Thinking about a national database of outcomes for people being seen under the Australian Government's Hearing Services Voucher Program, how important is each purpose listed below?  A national outcomes database also has some potential negative effects. How important do you think each negative effect listed below is to consider in the design of such a system? |

Table 7

Potential purposes of a national outcomes database

| Statement | Professional Consensus Percentage | Consumer Consensus Percentage |
| --- | --- | --- |
| To understand the impact of hearing loss on partners, family members and friends | **100%** | **89%** |
| To promote person-centred hearing care | **100%** | **93%** |
| To provide a national standard for effective hearing care services | **93%** | **95%** |
| To help determine best practice in hearing care | **97%** | **95%** |
| To enable hearing care professionals to compare their performance with national standards | **93%** | **86%** |
| To help researchers and hearing care professionals promote evidence-based hearing care | **93%** | **95%** |
| To help hearing care organisations know that they are providing high quality services | **93%** | **86%** |
| To provide information to the public about expected results from hearing care intervention | **93%** | **81%** |
| To help the Government develop national hearing care policy | **90%** | **98%** |
| To help hearing care professionals recommend hearing care options to clients | **86%** | **95%** |
| To help doctors and other health professionals support people with hearing loss | **86%** | **95%** |
| To help the Government target funding to hearing care services that achieve better outcomes | **86%** | **89%** |
| To provide evidence of the effective use of Government resources | **86%** | **86%** |
| To help researchers better understand hearing loss and hearing care | **83%** | **96%** |
| To help identify future needs of people with hearing loss | **83%** | **89%** |
| To help hearing care organisations identify which hearing care professionals are providing high quality services | 79% | **88%** |
| To help Government and other funders identify and audit hearing care organisations that are achieving poor outcomes | 79% | **91%** |
| To identify populations at risk for hearing loss | 76% | 77% |
| To help clients choose a hearing care organisation | 73% | **89%** |
| To help identify future trends in hearing science | 72% | **88%** |
| To help clients understand the evidence for hearing care | 70% | **88%** |
| To help researchers understand other medical conditions that are associated with hearing loss | 69% | **91%** |
| To enable a person with hearing loss to understand how their outcomes compare with outcomes of other people | 67% | 77% |
| To provide information about who in Australia has hearing loss | 66% | 72% |
| To rank hearing care organisations based on their outcomes | 34% | 77% |

Table 8

Potential concerns regarding the establishment of a national outcomes database

| Statement | Professional Consensus Percentage | Consumer Consensus Percentage |
| --- | --- | --- |
| Hearing care organisations could tamper with the data for more favourable results | **97%** | **89%** |
| Measures used could be inaccurate or measure the wrong thing | **97%** | **82%** |
| Hearing care professionals could tamper with the data for more favourable results | **93%** | **89%** |
| Results could be misused by competitors to make other hearing care organisations appear unfavourable to potential clients | **90%** | **82%** |
| A data breach could result in the release of personal information | **90%** | 75% |
| Poor outcome scores could be used to justify funding cuts | **90%** | 75% |
| Poor outcomes could remain despite poor results in the database | **83%** | 80% |
| Data could be misinterpreted | 79% | **84%** |
| Funding used for the database could detract from the funding available for hearing care services | 79% | 70% |
| Smaller hearing care organisations could be at a disadvantage due to fewer outcome results reported | 79% | 63% |
| Incomplete data could make the results unreliable | 76% | **89%** |
| Results could reflect poorly on a hearing care professional | 69% | 57% |
| Results could reflect poorly on a hearing care organisation | 62% | 43% |
| Hearing care professionals could spend too much time collecting outcome information | 59% | 48% |
| Hearing care organisations could be ranked based on their outcomes | 55% | 50% |
| Clients could be uncomfortable with being monitored | 55% | 41% |
| Hearing care organisations could incur administrative costs when collecting outcomes | 41% | 36% |

Table 9

Ranking of potential stakeholders to run a national outcomes database among the Consumer group

| Potential stakeholder | Consumer Consensus Ranking |
| --- | --- |
| Independent Research Organisation (e.g. NAL, CSIRO) | 1 |
| Professional organisations for hearing services (e.g. Audiology Australia, the Australian College of Audiology) | 2 |
| University Department | 3 |
| Australian Government | 4 |
| Hearing service companies | 5 |
| Hearing aid companies | 6 |

Table 10

Domains as presented to the consensus workshop

| Domain | Original Domain(s) |
| --- | --- |
| Improved communication (with others, with family, in groups) | “I can communicate effectively with people” (Consumer)  “I can communicate well with my family” (Consumer)  “Improved communication ability” (Professional)  “Improved communication in groups” (Professional) |
| Increased independence | “I can live my life independently” (Consumer) |
| Improved perception of clarity | “I hear clearly with my hearing aids” (Consumer) |
| Improved participation in activities | “I am able to do the things that I want to do” (Consumer) |
| Improved personal relationships | “Improved personal relationships” (Professional) |
| Improved self-management ability | “Improved self-management ability” (Professional) |
| Improved well-being | “Improved well-being” (Professional) |

Table 11

Recommendations made as a result of this project

| Aim 1: To seek views and consensus from a range of key stakeholders to define which client-centred outcome domains and measures should be used, when they should be used, and how they should be delivered, in a national Australian publicly funded hearing rehabilitation scheme. | |
| --- | --- |
| Recommendation 1: Target the outcome domains “communication ability”, “well-being”, “personal relationships”, and “participation restriction” |  |
| Recommendation 1a: Target the outcome domain “communication ability | - The ability for a person to engage in interpersonal communication was the most highly-ranked outcome domain. - Measures selected for the assessment of communication ability (i.e. conversation) should assess communication in general, but should also assess the two specific areas identified by stakeholders: communication with family, and communication in groups. - Part 2 of the GHABP targets communication ability and has been validated for use in device-focused hearing rehabilitation. It also includes a measure of device satisfaction. - The authors suggest that use of Part 2 of the four pre-specified situations of the GHABP will serve as an appropriate and relevant measure of communication ability in adult hearing rehabilitation. - It should be noted that this measure is specific to rehabilitation centred around the provision of hearing devices. Should non-device related rehabilitation need to be assessed, a measure of communication ability will need to be developed and validated in non-device related rehabilitation. |
| Recommendation 1b: Target the outcome domain “well-being” | - General well-being is likely to capture effects of hearing rehabilitation that may not be captured by other more specific measures of rehabilitation. - The use of a standardised well-being measure will facilitate the comparison of the well-being of adults receiving hearing rehabilitation with that of adults in general, and that of adults receiving treatment for other health conditions. - This will also facilitate the comparison of those adults who are fitted with hearing aids as part of their rehabilitation program with those who do not. - There is currently no clear measure of well-being that has been validated in hearing rehabilitation. - The authors suggest that a short question set that assesses well-being should be developed and validated in hearing rehabilitation. - The Warwick-Edinburgh Mental Well-Being Scale (WEMWBS) has been validated using robust psychometric methods and may serve as a useful starting point for this process. Targeting the short question set to hearing-specific well-being may also provide a more sensitive measure than a general well-being measure. |
| Recommendation 1c: Target the outcome domain “personal relationships” | - Hearing loss has significant impacts on the ability for people with hearing difficulty to interact with others and can have substantial negative effects on interpersonal relationships. - The impact of hearing loss on personal relationships was highly ranked by professional stakeholders, and the impacts of hearing loss on family was highly ranked by consumers of hearing services. - There is currently no good measure of the effects of hearing difficulty on personal relationships from the perspective of people with hearing difficulties. - The authors suggest that a measure should be developed to allow the measurement of the impact of hearing loss on personal relationships. - This may have the added benefit of being able to establish the effect of early rehabilitation on relationships, encouraging people experiencing hearing difficulties to seek early intervention and reducing ongoing disability |
| Recommendation 1d: Target the outcome domain “participation restrictions” | - The negative effects of hearing loss on people’s ability to participate in society are well-documented. - Increased participation in society has broad benefits both to society and to the individual. - The reduction of participation restrictions due to hearing rehabilitation should be targeted as a measure of the benefit of hearing services to society. - Existing measures of participation among people receiving hearing rehabilitation, such as the Social Participation Restrictions Questionnaire (SPaRQ), focus strongly on social participation, and do not include all aspects of participation. - The authors suggest that a short question set that assesses participation restriction should be developed and validated in hearing rehabilitation. - The SPaRQ is a research-quality measure of social participation, which has been validated using robust psychometric methods in hearing rehabilitation and may serve as a useful starting point for this process. However, additional items that assess non-social aspects of participation will likely need to be added to it. |
| **Aim 2: To identify current and future potential mechanisms and systems to standardise the collection of data and reporting of outcomes, to enable comparison across clients and hearing service providers.** | |
| Recommendation 2: Measure outcomes at baseline and then no earlier than three months following the conclusion of the rehabilitation program | - Obtaining outcomes at or before the fitting of hearing devices is likely to provide important information to support the rehabilitation process and establish the true impact of rehabilitation. A baseline measure should be established for adults receiving hearing rehabilitation. - There is evidence that hearing aid outcomes are likely to have stabilised by the three-month time point following rehabilitation. As a result, we recommend that outcomes following rehabilitation not be collected before this time point. - There is little evidence currently available regarding the stability of outcomes of hearing rehabilitation other than hearing aid fitting. - It is possible that some outcomes may take longer to stabilise for some people, and so ongoing review of the developed outcomes instruments should be conducted to ensure that they capture the effect of hearing rehabilitation programs. |
| Recommendation 3: Establish an independent body to develop a standardised outcomes instrument and mechanism for outcomes collection | - When asked who should collect outcomes, while there was very little agreement among professionals involved in the hearing industry, consumers had clear preference for collection and analysis of outcomes data to be conducted by a body perceived as independent from the hearing industry, such as universities, professional organisations, or other research organisations. - The establishment of an independent body to collect and analyse outcomes data has precedent in hearing rehabilitation in Sweden and in rehabilitation medicine in Australia. - The independent body should receive client data from hearing service providers or funders and contact clients for outcomes collection directly. - The independent body should be responsible for the ongoing development and management of an outcomes instrument to ensure that it is appropriately sensitive and specific while also minimising the burden on clients being contacted for outcomes collection. This may include the selection of items from existing measures to facilitate their use as part of the outcomes instrument. - The independent body should produce regular reports on client outcomes for funders of hearing services, the public, and hearing service providers. |

Figure 1

Flow of Delphi rounds for outcome domains section


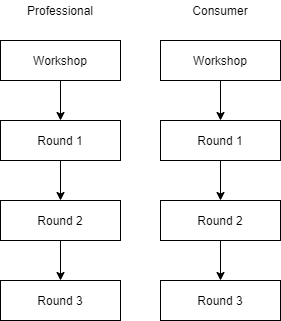


Figure 2

Flow of Delphi rounds for methods of collection and outcomes database sections


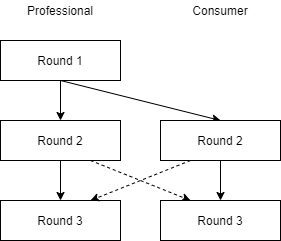


Figure 3

Flow of Delphi rounds for time of collection, reason for collection, and party responsible for collection sections


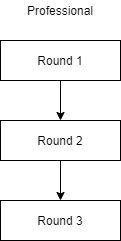

Supplement: Supplementary file 1 [file Data_Sheet_1.docx]
